# Implementation of learning into person-centred practice: evidence of impact from community nursing preparation programmes

**DOI:** 10.3389/frhs.2025.1598699

**Published:** 2025-08-08

**Authors:** Vaibhav Tyagi, Julie Churchill, Caroline Dickson

**Affiliations:** ^1^ Faculty of Medicine and Health, The University of Sydney, Sydney, NSW, Australia; ^2^ Division of Nursing and Paramedic Science, Queen Margaret University, Edinburgh, United Kingdom

**Keywords:** leadership, community nursing, education, person-centred curriculum, person-centred practice inventory

## Abstract

**Introduction:**

There has been a global move towards personalising and “humanising” healthcare and promoting caring cultures. Education is addressing this agenda by incorporating person-centred principles into teaching and learning. The aim of this research was to explore the implementation of person-centred learning into healthcare practice. More specifically, this study aims to explore community nurses' implementation of learning about person-centredness in their practice and to demonstrate the impact of person-centred curriculum.

**Methods:**

A cross-sectional quantitative survey design was used with community nursing graduates and current students who engaged with person-centred curricula.

**Results:**

Significant improvements were found in three constructs of person-centred practice—*clarity of beliefs and values, knowing self and developed interpersonal skills*.

**Discussion:**

These findings provide support for the development of pre-requisites of person-centred practice, rather than person-centred processes in pre-registration curricula. With key pre-requisites for person-centred practice such as leadership attributes of knowing self and of advanced communication skills, learners and graduates will be able adopt healthful leadership practices which are vital in developing others and in creating person-centred cultures.

## Introduction

Following the World Health Organisation's (1) commitment to placing people at the centre of healthcare, there has been a shift in the focus of health and social care systems globally. This shift is concerned with humanising healthcare where human rights principles such as dignity; respect for diversity and non-discrimination, accessibility, and equity; involvement and participation; partnership and empowerment are adopted as core values (2). According to McCormack and McCance (3), these principles reflect person-centredness. Current professional standards in nursing have responded to the WHO's agenda by moving from a technical focus in their standards to a stance that incorporates person-centred principles (4–6), although the challenge for curriculum leaders is operationalising these standards (7–13).

Despite these developments, it is reported that person-centred principles were not consistently applied in education curriculum; rather, they reflected heuristics prepared without a solid theoretical foundation of person-centredness (14, 15). In response to these challenges, a Person-centred Curriculum framework (PCCf) was developed with leaders and practitioners from education and practice (16). The framework presents as an open system, rather than an educational programme, and considers the centrality of shared values, the strategy, systems, and structure of the curriculum as well as leadership style, staff competence and capability (17). Consequently, there is a growing body of evidence that offers insight into person-centred practitioners' learning and leadership (16, 18–22). There is, however, a limited understanding of the sustainability of knowledge implementation post-graduation.

There is increasing global recognition of the importance of preparing healthcare professionals to deliver person-centred care (PCC), yet many programmes still lack consistent integration of PCC pedagogies (20, 23). Literature suggests that while curricula may include elements of PCC, these are often fragmented or under-theorised (14). Cardiff et al. (24) and Lynch et al. (25) emphasise that embedding reflective and relational components like “knowing self” fosters leadership and sustainable person-centred cultures. Furthermore, Heron's (26) facilitation theory and Dewing et al.'s (24) work on flourishing workplaces underline that learning environments must mirror the person-centred values they seek to instil. Despite promising models, there remains a gap in longitudinal evidence assessing the transition from person-centred learning to person-centred practice (21, 22).

**Figure 1:**
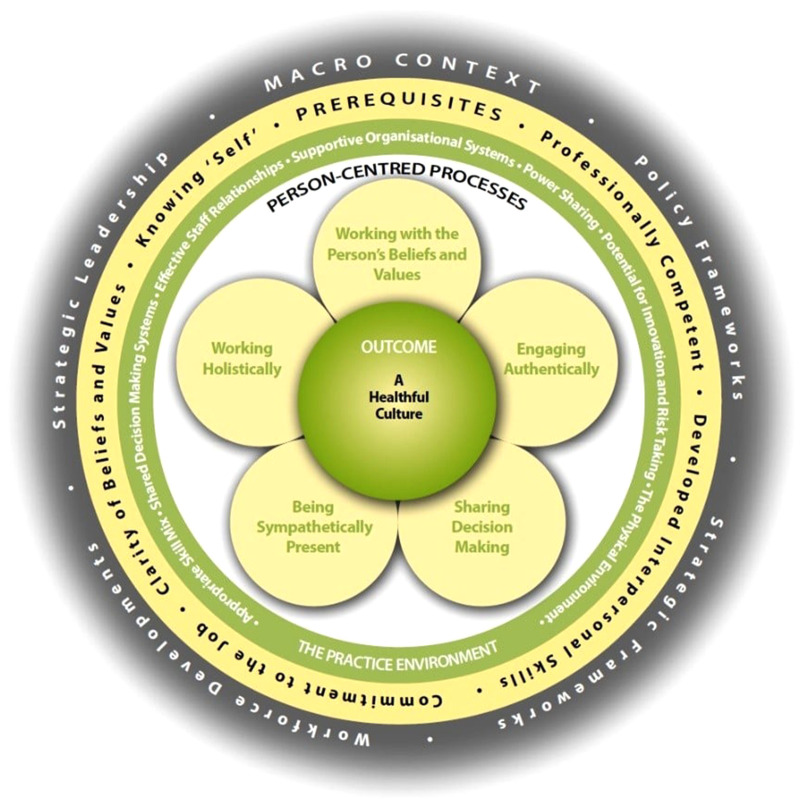
The person-centred practice framework (28).

The purpose of this research was to explore the implementation of person-centred learning into healthcare practice by community nurses. The aim was to explore long-term changes to the knowledge and practice of person-centredness in graduates compared to students on the programmes. We hypothesised that there would be significant positive changes in the knowledge and practice of person-centredness in community nursing graduates compared to the students on the programme. We further hypothesised, based on the nature of content and approaches within these nursing programmes that the changes would be prominent in the following domains of person-centred practice, as defined in the Person-centred practice framework (Figure 1) –

i. *Knowing self* and *Developed inter-personal skills* (Pre-requisites for person centred practice)
ii. *Shared decision-making systems* (Practice environment)

## Methods

The current study was conducted within three community nursing programmes—two within the Postgraduate Diploma in Person-centred Practice [Specialist Community Public Health Nursing (SCPHN)] and the Postgraduate Diploma in Person-centred Practice (District Nursing) (DN). These programmes reflect the PCCf and aim to develop leaders in community nursing. In the United Kingdom, SCPHNs are Health Visitors and School Nurses who form part of multi-professional care pathways supporting healthy pregnancy, and children aged 0–19 years while district Nurses play a key role in leading the integrated team in offering care and support to those whose needs are best met in a home setting.

### Design and sampling

A quantitative survey-based research design was used to explore implementation of learning into practice, specifically regarding developing person-centred culture and practice. The study received ethical approval from the Ethics committee at the authors' institution. An online version of the Person-centred Practice Inventory—Staff (PCPI-S) was deployed using Qualtrics online survey tool (29).

Purposive and convenience sampling was used. Graduates and part-time and full-time students were approached for participation and participant recruitment was facilitated through professional networks. Potential participants were briefed in online information sessions and a weblink to the online survey was provided. All participants were adults with the capacity to give informed consent, and there was no age restriction or exclusion based on other demographic variables. Consent was recorded on the first page of the online survey. Only after participants had clicked “agree” were they able to proceed to the survey.

### Data collection and analysis

PCPI-S is a standardised and psychometrically validated instrument (30) which consists of 17 constructs with 59 items in total. Each item asks participants to rate their agreement on a Likert scale of 1 (strongly disagree) to 5 (strongly agree). PCPI–S is a reliable instrument with high validity and is suitable for electronic distribution and data collection (30). Demographic data were also collected, namely age, sex, length of time since qualifying as a nurse, discipline, are they a student, if so what point of the programme were they at, number of years since qualifying from the programme, as was space for open comments to collect any other relevant information they wished to provide that may not have been captured in the PCPI-S e.g., information on current workplace, work environment, culture, and staff relationships.

Data from the survey were labelled, ID corrected and entered in a missing data analysis. This statistical analysis looked for discernible patterns of missingness and imputed missing data. Upon imputation, the data were entered in a Bayesian pairwise correlation analysis to explore the correlations between factors of interest. Factors of interest included domain and construct scores on PCPI-S, as well as specialisation and qualification of the participants (i.e., students vs. graduates). Demographic variables were entered as potential confounding variables in order to control their effects. Jeffrey's (31) suggestions were used to determine the statistical support for presence of a correlation (BF_10_ > 3 strong evidence, BF_10_ > 100 decisive etc.). Pearson's correlation coefficients were used to estimate the strength of the relationships among the variables. Finally, analyses of variance (ANOVAs) were conducted to examine the statistical difference between person-centred domain scores of current students and recent graduates. Statistical significance threshold was set at *p* < 0.05. All the analyses were conducted using R v 4.0.3 (32) and R Studio v 1.3.1093 (33). Bayesian correlation analyses and independent samples t-test were conducted using JASP (34).

Qualitative data from the open text questions were analysed using thematic analysis (35). This method served well to generate themes, identifying patterns of meaning. To undertake analysis, data were prepared by collating the text in table form and *familiarisation* was achieved by reading and re-reading the text. Initial codes were generated and checked by CD and JC. Through dialogue and debate, themes were generated, reviewed and then refined until the final themes were identified and named.

## Results

### Demographics

105 students enrolled on the programme at the time of the research and approximately 279 past graduates (from previous 5 years of the programmes) were approached for this study. 85 participants filled the survey, and 67 completed responses were retained. A summary of the participant demographics is provided in Table 1.

**Table 1:** Demographic characteristics of the sample.

Qualification	Qualified (*n* = 22)		Student (*n* = 35)		
Specialisation	qHV	qDN	sHV	sSN	sDN
N	14	8	13	9	13
Years since qualification (Avg)	3.14	1.62	–	–	–
Caseloads (Avg)	1.86	1.25	–	–	–
Sex (number of males)	0	2	0	0	0

Ten participants did not provide their demographic details. Specialisations were qHV, qualified specialist community public health nurse—health visitor; qDN, qualified specialist practitioner district nurse; sHV, student specialist community public health nurse—health visitor; sSN, student specialist community public health nurse—school nurse; sDN, student specialist practitioner district nurse.

### Quantitative findings

Specialisation and qualification (qHV, qDN, sHV, sDN & sSN) were entered as independent variables in a Bayes ANOVA with all the domains of the PCPI as dependent variables. Bayes ANOVA model with *Pre-requisites* domain showed statistically supported differences (BF_M_ = 38.8). Other domains of the PCPI did not show any statistically supported differences (*Care environment* BF_M_ = 1.93; *Care processes* BF_M_ = 2.26). *Post-hoc* comparisons across specialisations for *Pre-requisites* revealed statistically supported differences between qHV and sDN (uncorrected BF_10_ = 741, corrected posterior odds = 236.70) and qDN and sDN (uncorrected BF_10_ = 40.13, corrected posterior odds = 12.80).

Following this, individual constructs within the Pre-requisites domain were entered as dependent variables to tease out the nuances of these differences. Among these, *Developed interpersonal skills* (BF_M_ = 22.26), *Knowing self* (BF_M_ = 14.28) and *Clarity of beliefs and values* (BF_M_ = 23.11) showed statistically supported differences. Individual *post-hoc* comparisons for these are listed in Table 2.

**Table 2:** Individual *post-hoc* comparisons for constructs of the pre-requisites domain.

	Average differences in scores	Uncorrected BF10	Corrected posterior odds
Developed interpersonal skills			
qHV—sDN	0.59	60.50	19.33
qDN—sDN	0.60	7.92	2.53
sHV—sDN	0.50	4.48	1.43
Knowing self			
qHV—sSN	0.99	9.74	3.11
qHV—sDN	0.71	60.51	19.33
qDN—sDN	0.56	6.03	1.92
Clarity of beliefs and values			
qHV—sDN	0.73	14.36	13.85
qDN—sDN	0.92	46.57	14.88
sHV—sDN	0.59	13.28	4.24

### Qualitative findings

Three primary themes emerged from the qualitative responses: (1) Barriers within the practice environment, (2) Role-driven perceptions of agency, and (3) Emotional labour and moral tension.

1. Barriers within the Practice Environment

Participants across specialisations described a shared experience of under-resourced work environments, citing staff shortages, high caseloads, and systemic rigidity as major impediments to enacting person-centred practice:

“Constant demands due to understaffing due to a lack of staff and services has made the job difficult to manage and I am very stressed most of the time.”—Student District Nurse

“Large caseloads, limited protected time, staff shortages and lack of support are the main challenges within this role.”—Qualified Health Visitor

Emerging from the Covid-19 pandemic, the practice environment was described as a high stress environment featuring time constraints, understaffing, absenteeism, and lack of resources. This aligns with quantitative findings that showed no significant differences in the “practice environment” domain, suggesting that structural limitations may mute the implementation of person-centred values despite individual preparedness.

Other respondents perceived the practice context was not conducive to being person-centred augmenting the differences in the Pre-requisite domain. They emphasised the need to care for themselves, reflecting the construct of *Knowing self*:

“I also feel there should be more care and attention for the staff to have team building events to help to allow the staff working in very intense environments to destress and feel safe amongst their colleagues”—Student Health Visitor

Psychological distress, the perception of not being heard, and lack of respect and recognition were highlighted by one sDN and one qDN.

“I often feel self-care within teams is an issue. Staffing and burn out, stress levels all contributing to lack of respect for team members. I think we are person centred towards our patients and families but lack the same values within teams”—Student District Nurse

2. Role-Driven Perceptions of Agency

Students frequently reported feelings of powerlessness, highlighting their limited ability to challenge systemic barriers or initiate change:

“I feel I am not able to put what I have been taught on the DN course into practice due to lack of staff and time constraints.”—Qualified District Nurse

In contrast, some qualified participants described themselves as advocates and change agents, reflecting a greater sense of agency:

“I am an advocate for person-centred care! In my practice, with my team and often strive to encourage it at management level. The majority of my team feedback that they are well supported and enjoy my leadership style which involves treating them as the individuals they are”—Qualified District Nurse

This contrast supports the finding that development in “Knowing Self” and interpersonal skills (pre-requisites) was more pronounced in qualified professionals than students.

Responses were split into participants who perceived they had agency in being person-centred and those who did not.

“Sometimes it is difficult to deliver the care and attention to the child or young person that you would like to due to the lack of staff and resources available”—Student Health Visitor

Qualified nurses described respecting individuality, adaptability, and supportiveness.

“Treating individuals in a person-centred approach in practice on a regular basis is rewarding and essential”—Qualified District Nurse

3. Emotional Labour and Moral Tension

Many participants described a tension between their internalised values and the realities of practice, reflecting moral distress and a sense of loss when unable to practice person-centredness:

“There's guilt when you can't deliver care the way you were trained to. It weighs on you.” — Student Nurse

“I came from the CAMHS service which was very challenging emotionally. I value the person-centred approach because it recognises these emotional layers.”—Student Health Visitor

This underscores the emotional toll of person-centred care in unsupportive environments, aligning with literature on emotional labour in healthcare.

## Discussion

Findings of this study confirmed our first hypothesis which are consistent with in-house programme evaluations and pre-registration curricula grounded in person-centredness (10–12). Post-registration programmes in this study were effective in developing and sustaining knowledge implementation of person-centredness demonstrating significant differences in the domains of the Person-centred Practice Framework (PCPF). Application of the PCPF helps practitioners apply principles of person-centredness in practice, consistent with the framework aims (16–18).

Whilst the findings of Cook et al. (10) reported the development of pre-registration nurses' caring attributes (person-centred processes), the current study did not demonstrate these changes. Person-centred processes are, according to McCormack and McCance (3) the ways in which learners and practitioners engage with others. These processes have the intention of creating connections between persons and include working with the person's beliefs and values; being sympathetically present; engaging authentically; working holistically; and sharing decision-making. As Cook et al. (10) contend, these attributes are developed in pre-registration programmes, so it is perhaps unsurprising that post-registration learners in this study did not show development in this domain.

Findings of the current research show significant positive changes in the knowledge and practice of person-centredness in graduates compared to the post-registration students specifically in the pre-requisites domain of person-centredness (3, 20). Consistent with our second hypothesis, learners experience most significant development around the pre-requisites domain of the PCPF, particularly around the constructs of “knowing self” and and their “developed interpersonal skills”. There is a growing body of evidence to suggest “knowing self” is a key leadership attribute that contributes to the creation of healthful cultures (23, 24). In Cardiff et al.'s (24) model of person-centred leadership, “knowing self” is a precursor to engage authentically and compassionately with associates. By adopting relational practices such as “presencing”, “sensing”, “balancing”, “communing”, and “contextualising”.

Inconsistent with our final hypothesis, the results did not demonstrate notable changes within the practice environment domain of the PCPF, although thematic analysis gave some insight into the impact of context. The qualitative findings reinforce the critical interplay between individual readiness and environmental receptiveness. While learners developed intrapersonal attributes essential to person-centredness—such as reflective self-awareness and interpersonal skills—the practice environment often failed to scaffold or reward these attributes. The pervasive references to burnout, resource constraints, and feeling undervalued mirror existing research on moral injury and dissonance in nursing (13, 15, 24, 36–38). Notably, while students described frustration and helplessness, qualified professionals more often articulated a proactive, leadership-driven stance. This may reflect both their increased seniority and accumulated confidence, as well as the impact of the post-registration curriculum.

While learners gained skills such as reflective awareness and communication, systemic constraints often limited their enactment. These results echo Heron's (26) assertion that transformational learning must be situated within cultures that enable facilitation, not just instruction. If the curriculum fosters person-centred values but the clinical setting inhibits their expression, the outcome is often cognitive-affective dissonance. As one participant summarised:

“We are person-centred towards our patients and families but lack the same values within teams.”—Student District Nurse

This points to an under-addressed but critical facet of person-centred culture: intra-team dynamics. Internal team respect and psychological safety are prerequisites for delivering genuinely person-centred care externally. These findings also point to a dual responsibility: educators must cultivate intrapersonal development, and healthcare systems must evolve to support relational practices at scale.

### Implications for practice

Future iterations of person-centred curricula should explicitly bridge the gap between educational ideals and systemic implementation. Strategies may include: embedding simulation-based training focused on managing moral distress; equipping students with negotiation and advocacy skills; and supporting practice educators to role-model person-centred leadership within hierarchical systems.

As McCormack et al. (17) argue, system-level alignment is key. Educators cannot shoulder the burden alone—organisational leaders must partner to ensure that the workplace is not just a site of care delivery, but a co-facilitator of cultural change. Future implementation of person-centred curricula must be complemented by structural supports in practice settings. Protected time for reflection, recognition of emotional labour, and mentorship from person-centred leaders could help bridge the theory-practice gap. Embedding PCC not just in curriculum but also in institutional culture is essential for sustainability, as highlighted by Dewing et al. (24) and McCormack et al. (17). Further research could examine interventions where educational-practice alignment has led to measurable cultural shifts.

### Limitations

Despite a rigorous recruitment campaign, the study achieved a moderate sample size (*n* = 67), with a response rate of approximately 79% from those who accessed the survey. This limits the generalisability of the findings, particularly given the diversity of roles, settings, and healthcare systems within which community nurses operate. Although efforts were made to ensure representation across different specialisations (e.g., Health Visiting, School Nursing, and District Nursing), the sample may not fully reflect the broader population of community nurses, particularly those practicing in varied institutional or regional contexts beyond the study sites. Furthermore, the reliance on self-reported data introduces potential response bias, as individuals who felt strongly (positively or negatively) about their experiences may have been more likely to participate.

The low overall participation rate relative to the total number of eligible graduates and students (*n* = 384 approached) could be attributed to several factors, including the perception that the study was evaluative of one's professional competence or learning, as well as the known challenges of research participation in practice-based professions, where staff face significant workload pressures and limited time for non-clinical activities. These constraints likely skew the sample toward those with a higher degree of professional reflection or institutional engagement, potentially limiting the variability of responses. Additionally, the study's focus on a single national context (UK) further limits international transferability, particularly to systems with different nursing education structures or community health policies.

Therefore, while the results provide valuable insight into the impact of person-centred curricula, they should be interpreted as exploratory and context-bound. Future research should aim to replicate these findings using larger, more diverse, and ideally longitudinal samples to examine the sustainability of learning transfer into practice across time and setting. Mixed-method or multi-site designs that include objective indicators of practice environment and leadership context may also enhance the robustness and applicability of future evaluations.

## Conclusion

Current professional standards in nursing are moving from a technical focus to more person-centred principles in response to changes in WHO's policy commitments. The aim of this study was to evaluate the implementation of person-centred learning that is applied and sustained in practice. This study provides evidence that person-centred nursing programmes create an environment which allows the students to develop their pre-requisites for person-centred practice. Educators must encourage reflexive principles such as knowing self and clarity of beliefs and values to develop interpersonal skills in programme content. Furthermore, it is evident that practice educators and leaders need to provide more supportive environments where students and graduates feel able to practice person-centredness and promote person-centred ways of working.

## Data Availability

The raw data supporting the conclusions of this article will be made available by the authors, without undue reservation.
